# Efficiency enhancement with chloride to iodide ion exchange of benzimidazolium salt as a redox mediator

**DOI:** 10.3906/kim-2006-25

**Published:** 2021-04-28

**Authors:** Serkan DAYAN, Mert Olgun KARATAŞ, Bülent ALICI, Nilgün KALAYCIOĞLU ÖZPOZAN

**Affiliations:** 1 Drug Application and Research Center, Erciyes University, Kayseri Turkey; 2 Department of Chemistry, Faculty of Science, İnönü University, Malatya Turkey; 3 Department of Chemistry, Faculty of Science, Erciyes University, Kayseri Turkey

**Keywords:** Benzimidazole, DSSC, photovoltaics, dye-sensitized solar cell

## Abstract

The new benzimidazolium derivative (SM-1) salt with ion exchange from the (SM-0) was fabricated and characterized by proton-nuclear magnetic resonance (^1^H-NMR), carbon-nuclear magnetic resonance (^13^C-NMR), Fourier-transform infrared spectroscopy (FT-IR), electrospray ionization (EIS-MS), thermal analysis (TG), cyclic voltammetry (CV), and ultraviolet-visible spectroscopy (UV-vis), for electrolytes (liquid or dried) in the DSSC charge transportation mechanism. Also, the influence of ion exchange from chloride to iodine in the synthesized electrolytes, compared to other electrolytes (conventional or commercial), was investigated about DSSC performance efficiency. When using as a liquid electrolyte (SM-1), the power conversion efficiency (ƞ) of the working DSSC device was recorded as 1.980% and it was observed that the performances of DSSCs increased up to 56% when comparing dried electrolyte for SM-1 without conventional redox material (I^-^/I_3_^-^). In the future, different molecular modifications of this type of benzimidazole derivatives or mixtures with conventional redox couples may further improve the performance of DSSC devices.

## 1. Introduction

Dye-sensitized solar cells (DSSCs), which consist of photoanode, photoactive paint, electrolyte, and counter electrode main materials, are called the new generation and attract attention with their low cost and easy production ability [1–4]. However, the most significant problem with DSSC devices is seen as stable high performance, and much research is being carried out to overcome this negative situation [5–8]. The studies that are carried out by producing modifications of the main materials still maintain a high level of interest in this subject. The importance of electrolytes is as high as the photoactive layer and counter electrode in the DSSCs mechanism and the performance of these electrolytes, which are critical to the successful completion of the charge transfer process and the continuity of the electron current, directly affect device performance [9]. The most commonly used DSSC electrolyte is the iodide/triiodide (I^-^/I_3_^-^) redox couple and the main reason for the use of this redox couple is based on the rapid functioning of its kinetics within the DSSC mechanism [10]. When these parameters are examined, it is seen that the electron injection of the TiO2 metal oxide coating into the conductivity band takes place in femtoseconds (1 femtosecond (fs) = 10^-15^ s). This process occurs much faster in the combination of triiodide and the electron, as the oxidized dye molecule reacts with iodide without being combined with the injected electrons. In the electrolyte, triiodide diffuses in the cathode to collect electrons and undergoes iodide formation to transfer electrons to dye molecules.

By adding organic-based structures or salts such as 4-tert-butylpyridine [11], guanidinium isothiocyanate [12], methyl-benzimidazole [13] as an additional additive component to the traditional iodide/triiodide (I^-^/I_3_^-^) redox couple used in the literature studies, performance improvement studies were carried out. Thus, the conductivity of this conventional electrolyte (I^-^/I_3_^-^) can be increased from the outside with additional salts, especially in recent years significant studies have been achieved by adding salt structures containing imidazole rings to the electrolyte solution [9,14–16]. Successful results have also been achieved with solid electrolytes, in which imidazole-derived compounds are used without additional additives or a redox couple [17,18]. The general opinion of these studies is that the presence of alkyl chains of imidazolium salts may have a significant effect on the performance. Thus, although these additive components are not directly involved in main photoelectrochemical processes, they can make positive contributions to performance efficiency and stability. Herein, we reported that the 1-methyl-3-((1,2,3-benzotriazole)methyl)-benzimidazolium iodide (SM-1) salt was fabricated with ionic exchange from chloride salt (SM-0) and used as electrolyte in dye-sensitized solar cell prototypes (DSSC), and attention has been paid to the performance advantage due to ion difference. Benzimidazoles or imidazoles and their derivatives can be used both as the direct electrolyte and also as the support electrolyte to allow charge transfer within the dye-sensitized solar cell (DSSC) mechanism through molecular modifications in the future.

## 2. Experimental 

### 2.1. Synthesis of 1-methyl-3-((1,2,3-benzotriazole)methyl)-benzimidazolium iodide (SM-1)

Stage 1: A ethanolic solution of the synthesized benzimidazole molecule (0.5 mmol, 10 mL) was added to the base (KOH, 0.5 mmol), which was used for deprotonation, and stirred for one hour at ambient temperature. After that, the methyl iodide was added to the mixture with controlled dropwise and heated/stirred overnight at 76 °C. The resulting mixture was diluted with pure water (30 mL), in order to neutralize the mixture and remove any impurities, and the extraction process was performed with chloroform three times (10 mL). The residue was washed again with pure water and dried over anhydrous magnesium sulfate (MgSO_4_) and distilled off and the 1-methyl-3-((1,2,3-benzotriazole)methyl)-benzimidazolium chloride (SM-0) molecule was obtained.

**Figure 1:**
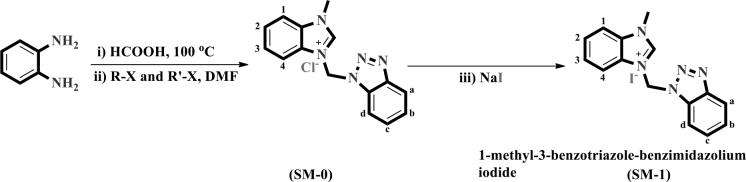
Synthesis scheme of SM-0 and 1-methyl-3-((1,2,3-benzotriazole)methyl)-benzimidazolium iodide (SM-1).

Stage 2: A solution of the fabricated SM-0 (DMSO/MeOH, v/v:3/1, 15 mL) (0.2 mmol) was added to the sodium iodide (0.25 mmol), which for halide exchange, and stirred overnight at 40 °C, after cooling to ambient temperature, the volatile of the resulting mixture was removed under vacuum system to dryness. The residue was washed with cooled pure water and methyl alcohol (three times 5 mL). The pure product SM-1 was dried under a reduced atmosphere (Figure 1). This ion exchange process increases the solubility of the molecule for electron transfer in the DSSC mechanism.

####  2.1.1. Data for 1-methyl-3-((1,2,3-benzotriazole)methyl)-benzimidazolium chloride (SM-0).

Color: light yellow. Yield: 65%. FW: 299.75 g/mol. ^1^H-NMR (DMSO, *δ *ppm): 4.11 (s, 3H, -C*H3*), 7.48 (t, ^1^H, -*Hb*), 7.67–7.79 (m, 5H, -*Hc*, -*H2,3,* R-C*H2*-R’), 8.02 (d, ^1^H, -*H4*), 8.11 (d, ^1^H, -*Ha*), 8.32 (d, ^1^H, -*Hd*), 8.45 (d, ^1^H, -*H1*), 10.55 (s, ^1^H, -N-C*H*=N+-). ^13^C-NMR (DMSO, ppm): 33.7 (-*C*H3), 55.9 (R-*C*H2-R’), 111.1, 113.8, 119.5, 124.9, 124.9, 126.9, 127.2, 128.6, 130.1, 131.9, 132.4, 143.8, 145.1 (-N-*C*H=N+-). 

#### 2.1.2. Data for 1-methyl-3-((1,2,3-benzotriazole)methyl)-benzimidazolium iodide (SM-1)

Color: yellow. Yield: > 90%. FW: 391.20 g/mol. ^1^H-NMR (CDCl3, δ ppm): 4.29 (s, 3H, -C*H3*), 7.42 (t, ^1^H, -*Hb*), 7.64–7.73 (m, 4H, -*Hc*, -*H2-4*), 7.84 (s, 2H, R-C*H2*-R’), 8.02 (d, ^1^H, -*Ha*), 8.35 (d, ^1^H, -*Hd*), 8.72 (d, ^1^H, -*H1*), 11.89 (s, ^1^H, -N-C*H*=N+-). ^13^C-NMR (CDCl3, ppm): 34.5 (-*C*H3), 57.3 (R-*C*H2-R’), 111.1, 112.8, 114.7, 120.2, 125.3, 128.1, 128.5, 129.7, 130.7, 131.8, 132.3, 142.6, 146.0 (-N-*C*H=N+-). IR (cm–1): 3113, 3053, 3002, 2977, 2926, 2894, 2852, 2756, 1611, 1557, 1511, 1489, 1477, 1452, 1436, 1422, 1385, 1367, 1336, 1307, 1291, 1251, 1228, 1205, 1192, 1155, 1139, 1128, 1103, 1018, 977, 959, 909, 881, 864, 853, 812, 782, 758, 742, 702, 666, 646, 622, 616, 608, 597, 576, 565, 539, 533, 524, 513, 502, 483, 469, 455. UV–vis (l(nm), (ε (L/(mol.cm))): 304 (38709), 356 (21104). Positive ESI^-^MS (m/z): 264.20 (calc: 264.30).

### 2.2. Photovoltaic measurements for DSSC devices 

The preparation materials for photovoltaic measurements are as follows:

· Gaskets, sealing caps, and masks.

· The platinum counter electrode (FTO coated glass, drilled for electrolyte, 1 cm2 active area).

· Titania electrode (FTO coated glass, 0.36 cm2 active area) by Solaronix SA (Aubonne, Switzerland).

· Ruthenium Dye Z907 (cis-Bis(isothiocyanato)(2,2′-bipyridyl-4,4′-dicarboxylato)(4,4′-di-nonyl-2′-bipyridyl)ruthenium(II)).

**Figure 2:**
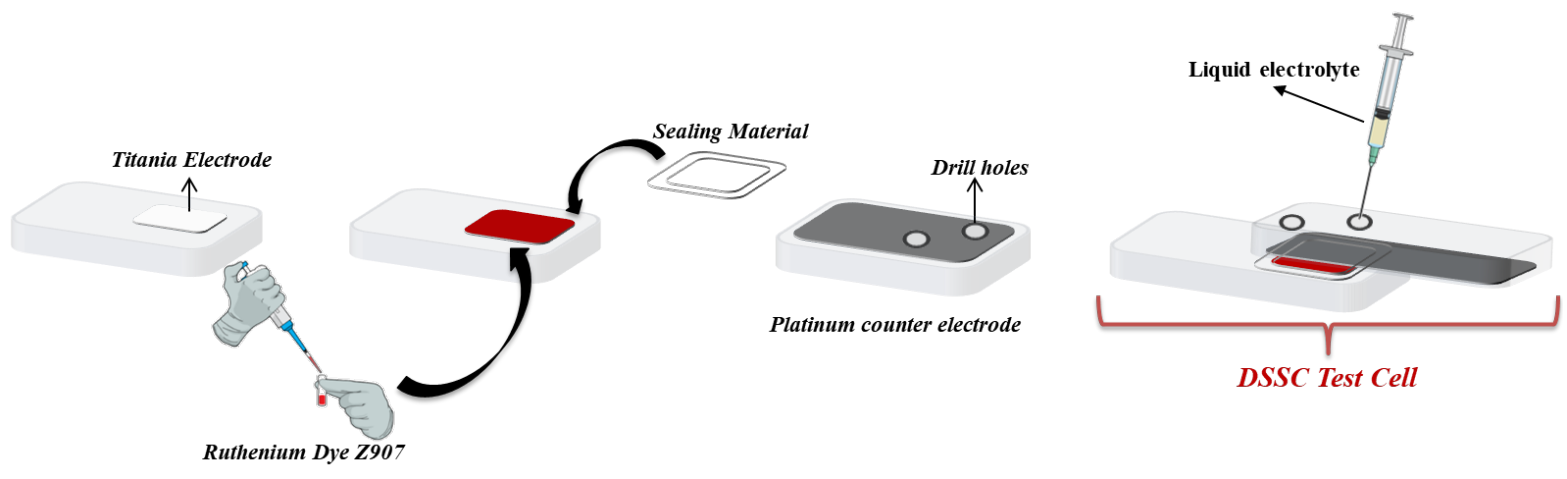
The fabrication dye-sensitized solar cell (DSSC) devices.

The TiO2 photoelectrodes were immersed into the ruthenium dye Z907 (1 × 10–3M, DMSO) within 24 h at ambient temperature to complete adsorption. The photoactive dye-loaded photoanode layer as a working electrode and platinum-coated FTO as a counter electrode were separated by a sealing (hot-melt) material (Meltonix 1170-60, city, country?) and then the devices were sealed together by manually pressing (≈15 psi) them under hot-plate heat. The methanolic or DMSO/MeOH solution of the synthesized electrolytes (SM-0 and SM-1) (saturated solution), Iodolyte AN 50 (Solaronix SA), and traditional redox material I^-^/I_3_^-^ was injected repeatedly into the interspace between the working electrode (TiO2) and the counter electrode (Pt-coated FTO) via two holes on the counter electrode and dried on a hot-plate, approximately at 50 °C until the photoanode layer was filled with dried electrolyte SM-1. The holes were also sealed with hot-melt material covered with a glass slip under heat. The efficiency of the DSSC devices was recorded by the current density–voltage (*J-V*) curves with a Metrohm Autolab PGSTAT101 device under the illumination of a simulated AM 1.5 solar light. A black mask with an aperture area of 0.36 cm2 was used during measurement to avoid stray light (Figure 2). A crosssectional view of a representative sample of the fabricated DSSC was taken. In this image, the thicknesses of the layers were determined as ≈ 11.50 µm (TiO2 layer) and ≈ 4.00 µm (ruthenium photoactive layer and electrolyte layer) (Figure S1).

## 3. Results and discussion 

### 3.1. Characterization data

The synthesis routes of 1-methyl-3-((1,2,3-benzotriazole)methyl)-benzimidazolium iodide (SM-1) are demonstrated in Figure 1. 

**Figure 3:**
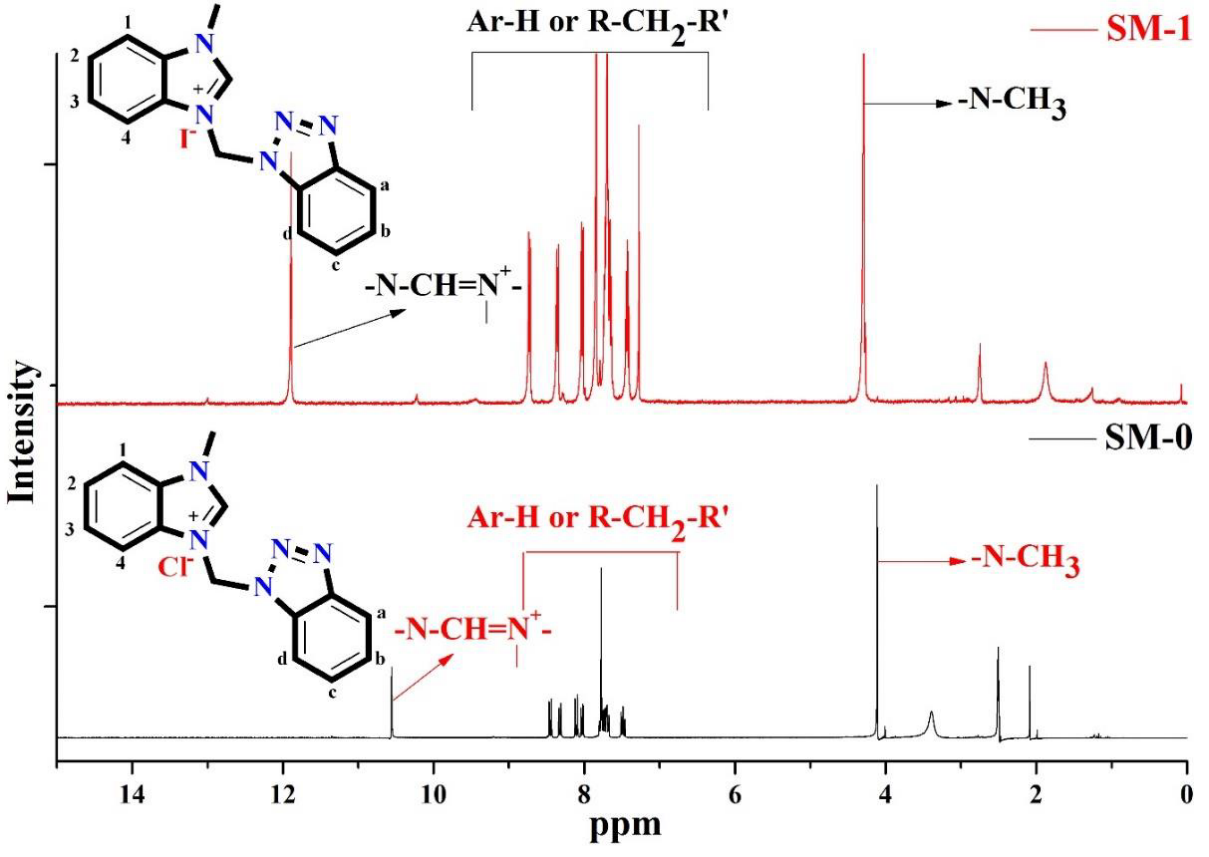
1H-NMR spectra comparison of SM-0 and SM-1 compounds.

In the ^1^H-NMR spectra of SM-0 and SM-1, the -C*H3* protons were assigned at δ = 4.11 ppm as a singlet peak for SM-0 and at δ = 4.29 ppm as a singlet peak for SM-1. Likewise, the -Ha, -Hb, -Hd, and -H1 protons which on the aromatic ring were located between 8.02–8.11 ppm as a doublet, 7.42–7.48 ppm as a triplet, 8.32–8.35 ppm as a doublet, and 8.45–8.72 ppm as a doublet, respectively. Also, the R-C*H2*-R’ protons that are attached to the benzotriazole ring were observed at around 7.67–7.84 ppm as singlet peaks and the carbene precursor -N-C*H*=N+- protons for SM-0 and SM-1 molecules were assigned at 10.55 ppm and 11.89 ppm as singlet peaks (Figure 3).

**Figure 4:**
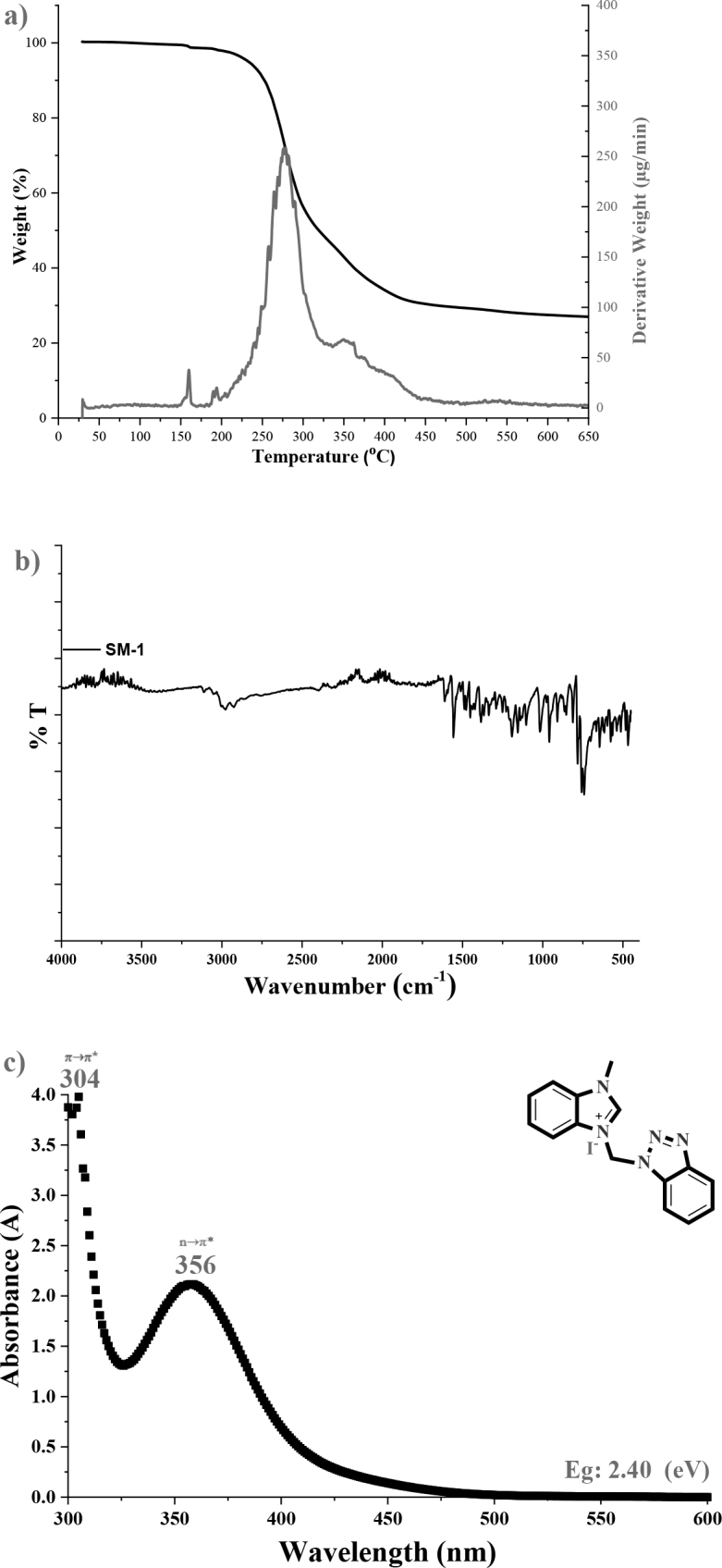
TG/DTG thermogram curve (a), FT-IR spectrum (b), and UV-vis spectra (1 × 10–4 M, solution in MeOH) of the SM-1 salt.

The synthesized SM-0 and SM-1 benzimidazoles exhibit ^13^C-NMR chemical shifts at δ = 145.1 and 146.0 ppm, respectively, for the characteristic -N-*C*H=N+- carbon atom, and the chemical shifts are comparable to those of other recorded carbene precursors (NHCs) or benzimidazoles [19,20]. Also, the -*C*H3 and R-*C*H2-R’ carbons were assigned at 33.7 and 34.5 ppm, and 55.9 and 57.3 ppm, respectively. It is evident from the characterization data obtained that when the chlorine ion and the iodine ion are replaced, the chemical shift values shift downfield (Figure S2 in the Supporting Information). Also, the FT-IR spectra for the SM-1 was shown in Figure 4a.

### 3.2. Thermal analysis

The thermogravimetric/derivative thermogravimetric (TG/DTG) curve (by Perkin Elmer Diamond, PerkinElmer, Inc., Waltham, MA, USA) of the SM-1 electrolyte is show in Figure 4b and the thermal decomposition steps were demonstrated in Table 1. 

**Table 1:** Thermal analysis results of the synthesized SM-1 salt.

Compound	Step	DTGmax (°C)	Ton (°C )	Tend (°C )	Mass loss (%)
SM-1	I	159.3	29.3	165.4	1.30
	II	276.5	165.4	451.3	68.32
	III	-	338.1	650.0	5.04

* Ton – thermal degradation onset temperature, Tmax – maximum weight loss temperature, Tend – final thermal degradation temperature.

When thermal analysis data is examined in detail, the synthesized SM-1 electrolyte decomposed with three steps at 50 °C to 650 °C**,** including both volatiles and organic residues. The thermal decomposition steps parameters demonstrate that the proportions in each stage for the residues were obtained as 98.70% for step I (residual solvents and initial decays), 30.38% for step II, and 25.34% for step III which calculated from the DTG curve. The thermal degradation onset temperatures of the SM-1 were recorded as 29.3, 165.4, and 338.1 °C, and the maximum mass loss temperature range of the compound was founded at between 165.4 to 451.3 °C in step II which represents the degradation of organic fragments. It is a positive situation for the SM-1 molecule to be limited to only volatile compounds up to 165 °C. This result is an advantage in terms of solar cells that get hot during operation.

### 3.3. Optical and electronic properties

The UV-vis spectrum of 1-methyl-3-((1,2,3-benzotriazole)methyl)-benzimidazolium iodide (SM-1) molecule in methyl alcohol (1 × 10–4 M) is exhibited in Figure 4c. The absorption spectra demonstrated that the n→π* and π→π* electronic transition peaks were recorded at 356 and 304 nm, respectively. From the spectrum, the bandgap energy (Eg), was calculated as 2.40 eV by the onset of the absorption spectra in MeOH with Eg = 1241/l onset formulation [3,21]. 

**Figure 5:**
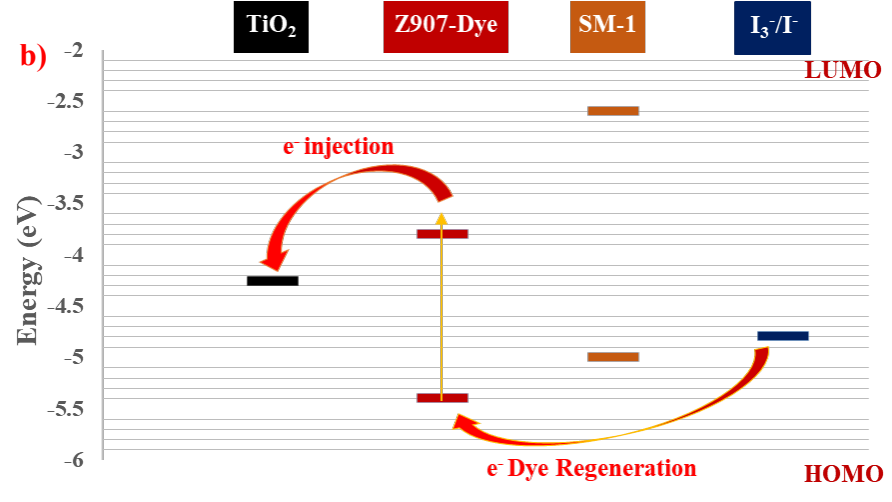
Cyclic voltammetry (CV) curve of the synthesized SM-1 salt at a scan rate of 20 mV s–1 and the schematic energy levels for electron mobility.

The highest occupied molecular orbital (HOMO) and lowest unoccupied molecular orbital (LUMO) energy levels of the SM-1 molecule are requisite data to identify the facilition of the redox cycle from the counter electrode to the photoactive excited dye. Cyclic voltammetry (CV) was performed with tetrabutylammonium perchlorate (TBAP) as a supporting electrolyte (0.1 M) in MeOH solution (Figure 5a). All redox potentials were referenced to the ferrocene/ferrocenium (Fc/Fc+) couple, which has a redox potential of __? (EFc/Fc+ = 0.510V versus Ag/AgCl?

The oxidation potential (Eoxd), EHOMO and ELUMO values of the synthesized SM-1 were calculated as 0.2 V, –5.00 eV and –2.60 eV from , EHOMO = –Eox + (–4.8) and ELUMO = EHOMO + Eg formulations [22] , respectively (Figure 5b).

### 3.4. Photovoltaic studies

Benzimidazole or imidazole-based salts are the most studied electrolytes (liquid or dried) due to their higher efficiency in dye-sensitized solar cells. The solar cell devices fabrication is mentioned in the photovoltaic measurements for DSSC devices. Herein, we exchanged the ion belonging to benzimidazole salt and tested an electrolyte in DSSC. From the optoelectronic parameters, the different DSSCs were fabricated with the aim of investigation of the electrolyte performance of the 1-methyl-3-((1,2,3-benzotriazole)methyl)-benzimidazolium iodide (SM-1).

**Figure 6:**
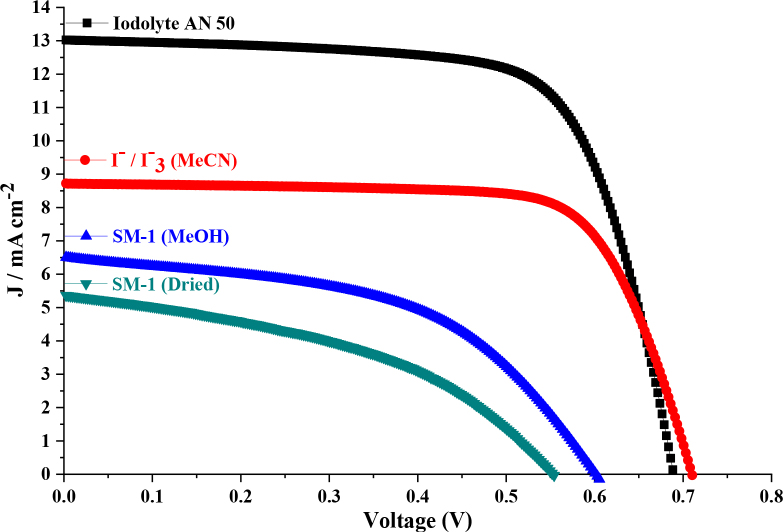
Current (J)–voltage (V) curves of DSSC devices consisting of different electrolytes.

The photovoltaic data and results including, fill factor (FF), short circuit current density (*Jsc*), open-circuit voltage (Voc), and all of the device’s efficiency (ƞ) are summarized in Table 2. Also, the recorded current (*J*)–voltage (*V*) curves as the average of three measurements are demonstrated in Figure 6, which include the performance of Iodolyte AN 50 (Solaronix) [3], I^-^/I_3_^-^, SM-0 (DMSO/MeOH) and SM-1 (dried and liquid). 

**Table 2:** Photovoltaic results of the fabricated DSSC devices.

Entry	Electrolyte	Jsca (mA/cm2)	Vocb (V)	FFc	ƞd (%)
1	SM-0 (DMSO/MeOH)	9.42×10–2	0.459	0.30	0.013
2	SM-1 (MeOH)	6.50	0.603	0.51	1.980
3	SM-1 (Dried)	5.36	0.554	0.42	1.270
4	I–/ I3– (MeCN)	9.04	0.710	0.69	4.490
5	Iodolyte AN 50	13.0	0.691	0.70	≈6.300

a Short-circuit current density, b Open-circuit voltage, c Fill factor, d Power conversion efficiency.

As a standard initial consideration, we planned to improve the performance of DSSC prototypes with simple ion exchange, and some studies carried out with similar ideas are included in the literature. We also compared the performances of the SM-1 electrolyte obtained by ion exchange and both the conventional I^-^/I_3_^-^ electrolyte and the improved commercially available Iodolyte AN 50 (Figure 6). It was also aimed to improve DSSC performance by eliminating the solubility problem. Also, when the cell performance of the SM-0 was examined, the power conversion efficiency (ƞ) was 0.013% as a result of both low solubility and lack of suitable molecular structure. Also, it was understood from the DSSC performance values that the SM-1 obtained by ion exchange and including iodine was suitable for the DSSC mechanism both in terms of solubility and molecular structure and its performance was studied as liquid and dried electrolyte in DSSC prototype. In terms of the photovoltaic performance results, the cell efficiency was recorded to be 1.980% (ƞ) when SM-1 was used in combination with methyl alcohol solvent, and the power conversion efficiency was recorded as 1.270% (ƞ) when used as a dried without any solvent (Table 2). When the performance data between the dried-state electrolyte and liquid state electrolyte were examined, all photovoltaic parameters *Jsc*, Voc, FF of the liquid state SM-1 electrolyte were higher and when compared with other standard electrolytes such I^-^/I_3_^-^, Iodolyte AN 50, under the same conditions, these performance values were promisingly good. Ionic molecules improved the power conversion efficiency and the charge transportation in the DSSC devices. However, due to the low values of short circuit current density (*Jsc*), molecular engineering studies will be carried out in the future. 

The DSSC devices parameters (Jsc, Voc, FF, and ƞ) are recorded as 6.50 mA/cm2, 0.603V, 0.51, and 1.980% for the best cell. There is about a 56% increase in performance compared to the dried results (Table 2). The improvement in DSSC performances with SM-1 can be attributed to the quasi-solid electrolyte [5]. Herein, when the recorded performance is compared with some studies in the literature, it is determined that the best cell performance in this study sheds light on further studies and has a potential (Table 3). In particular, a good performance was obtained when the SM-1 molecule was used as a quasi-solid electrolyte and it was predicted that better performance results could be obtained by producing analogs of this molecule. As can be seen in Table 3, it is possible to improve device performance by using different additive materials, and these results may differ according to the design of the materials

**Table 3:** The comparative data of the DSSC performance.

Entry	Electrolyte	Jsca (mA/cm2)	Vocb (V)	FFc	ƞd (%)	Reference
1	Imidazolium salts (compound 3)	11.48	0.550	0.42	2.650	4
2	Imidazolium salts (compound 4)	8.18	0.550	0.34	1.53	4
3	[C4BEP][BF4]2/DMPII/I2 (solid)	6.2	0.71	0.67	2.94	9
4	[C6BEP][TFSI]2/DMPII/MPII/I2 (solid)	12.33	0.81	0.60	6.02	9
5	PMIm (solid)	12.65	0.71	0.70	6.3	18
6	PMPi (solid)	5.21	0.66	0.65	2.2	18
7	DMPImI (solid)	0	0	0	0	18
8	SM-1 (MeOH)	6.50	0.603	0.51	1.980	Present study
9	SM-1 (dried)	5.36	0.554	0.42	1.270	Present study

## 4. Conclusion

Benzimidazolium or imidazolium salts in dye-sensitized solar cells (DSSCs), which include photoanode, photoactive dye, electrolyte, or redox mediator and counter electrode, are used as an electrolyte or support electrolyte. Herein, we designed the ion-exchanged benzimidazolium salt bearing methyl and benzotriazole fragment and highlighted the benefits of this change for charge transportation mechanism in the DSSC prototype. The efficiencies of the working devices changed depended on the liquid (1.980%) or dried (1.270%) electrolyte for SM-1 and the type of ion it contains and the redox system. Despite the efficiency enhance steps were accomplished for our devices, the overall performances remain too low against the commercial product Iodolyte AN 50 which containing the imidazolium salts, I^-^/I_3_^-^ etc. Production of easily available, cost-effective, applicable salts is important for DSSCs, and benzimidazole or imidazole-based salts are also critical for the development of DSSC cells. Redesigning the molecular structures of the corresponding salt will enable the production of high-performance devices in the future.

Supplementary Materials
