# Supplementary material for: Efficiency enhancement with chloride to iodide ion exchange of benzimidazolium salt as a redox mediator
Source: Turk J Chem. 2021 Apr 28;45(2):333–41. doi: 10.3906/kim-2006-25 (PMC8169510; doi:10.3906/kim-2006-25)
Supplement: Supplementary file 1 — Supplementary Materials [file turkjchem-45-333-sup001.pdf]

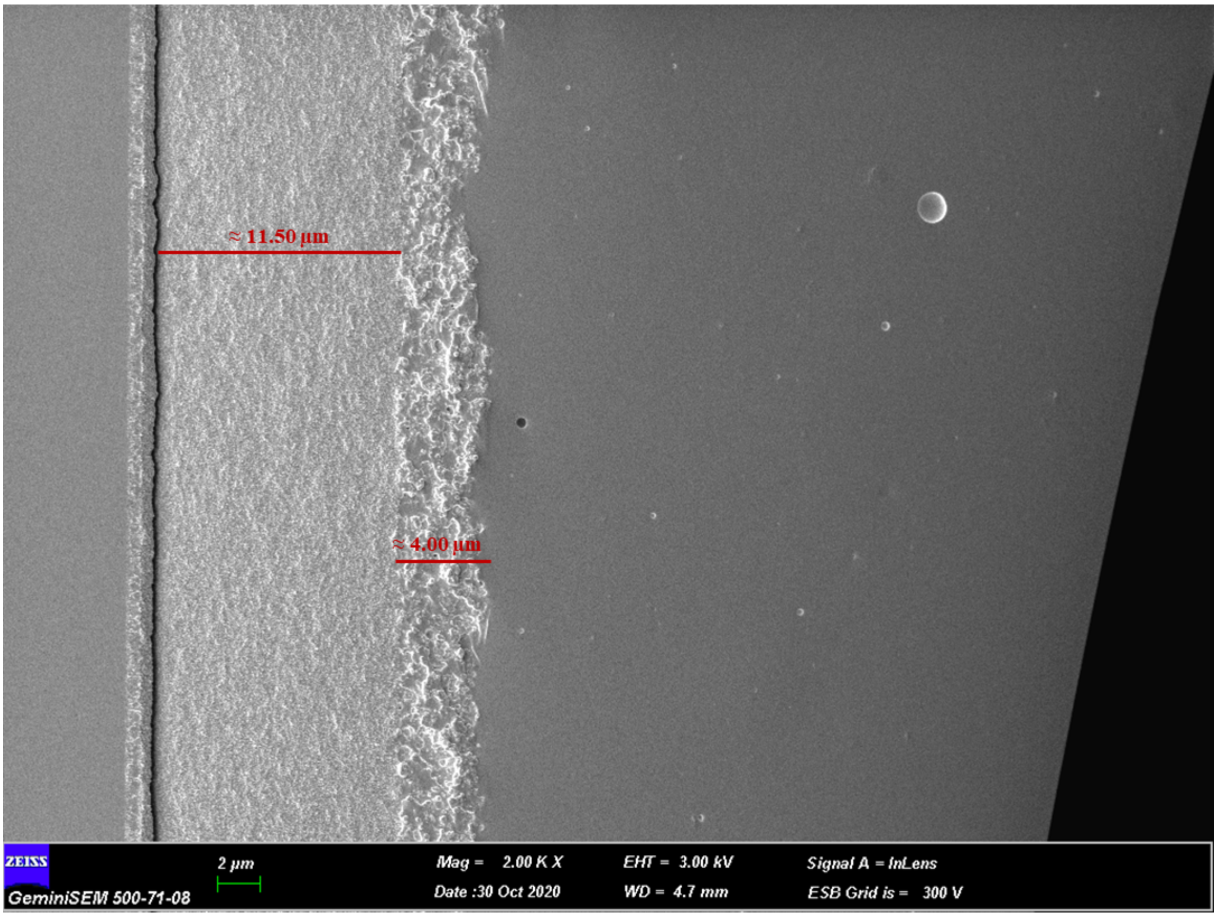

Figure S1. SEM analysis of a representative DSSC device.

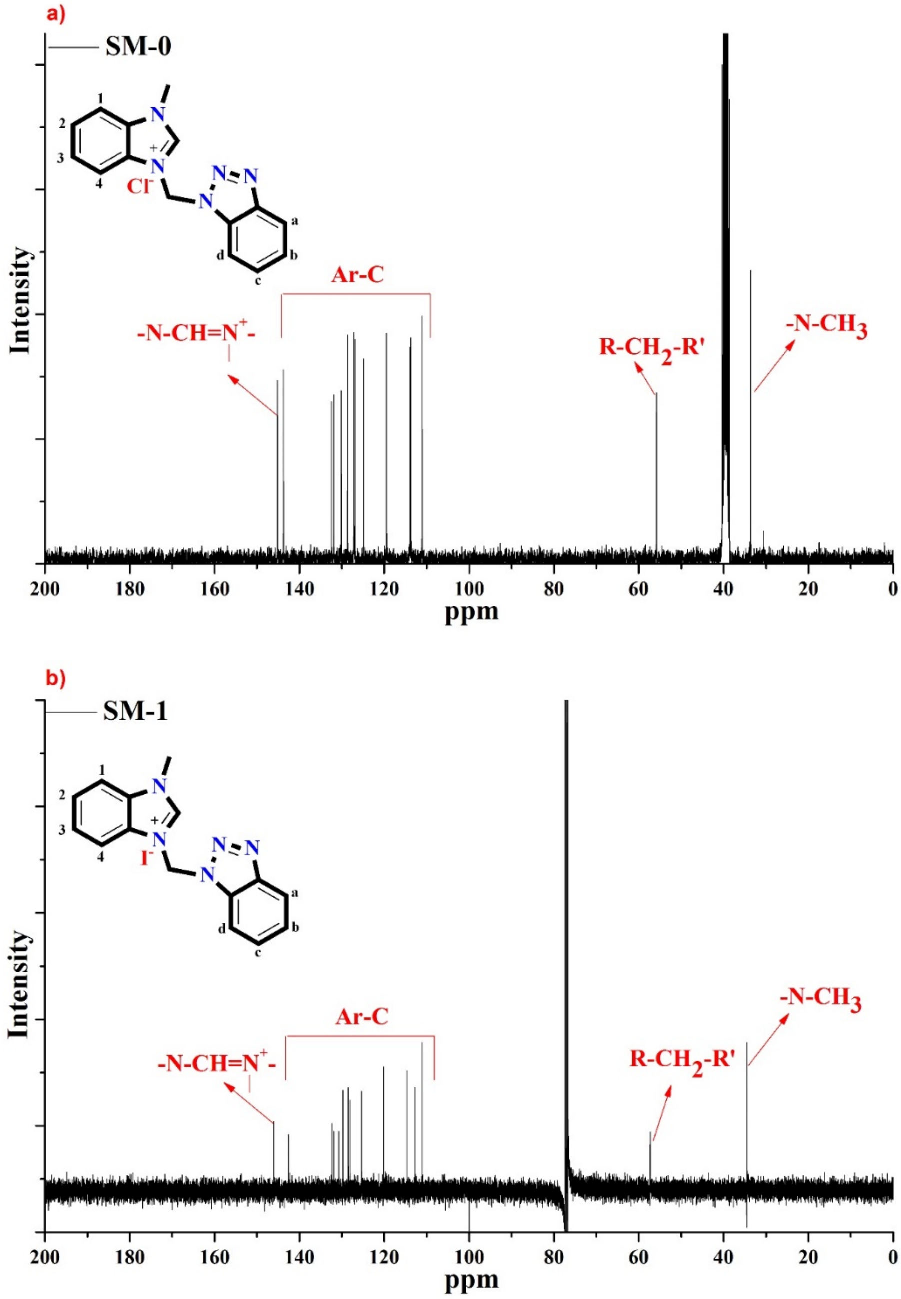

Figure S2. ^{13}C-NMR spectra comparison of SM-0 (a) and SM-1 (b).
